# Prevalence, socioeconomic factors and obstetric outcomes associated with adolescent motherhood in Ceará, Brazil: a population-based study

**DOI:** 10.1186/s12884-021-04088-7

**Published:** 2021-09-08

**Authors:** Shirley Kelly Bedê Bruno, Hermano Alexandre Lima Rocha, Sabrina Gabriele Maia Oliveira Rocha, David Augusto Batista Sá Araújo, Jocileide Sales Campos, Anamaria Cavalcante e Silva, Luciano Lima Correia

**Affiliations:** 1 State University of Ceará, Fortaleza, CE Brazil; 2 Department of Global Health and Population, Harvard T. H. Chan School of Public Health, Boston, MA USA; 3 Department of Maternal and Child Health, Federal University of Ceará, Rua Professor Costa Mendes, 1608 5 Andar, Fortaleza, Ceará Brasil CEP: 60430-140 Brazil; 4 Department of Community Health, Federal University of Ceará, Fortaleza, CE Brazil; 5 ISEC, University Center Unichristus, Fortaleza, CE Brazil

**Keywords:** Pregnancy in adolescence. Prevalence. Poverty. Health services. Obstetrics

## Abstract

**Background:**

Adolescent motherhood (AM) remains a public health problem, especially in low and middle income countries, where approximately 95% of these births occur. Evidence from studies with population representativeness about events associated with AM is limited. We assessed the prevalence of AM, as well as its association with Socioeconomic Factors and Obstetric Outcomes.

**Methods:**

A population-based cross-sectional study on maternal and child health of women aged 10 to 49 years, living in the state of Ceará, in northeastern Brazil was carried out to assess the prevalence of AM, as well as its association with Socioeconomic Factors and Obstetric Outcomes. The definition of adolescence used in the study was the one utilized by the WHO. In addition to the interview, data were double-checked according to the information in the government’s pregnancy health booklet. Sample-adjusted logistic models to determine the association of socioeconomic factors and AM, as well as the association of AM with obstetric outcomes, with a causal approach to multivariate analyses, were used.

**Results:**

The prevalence of adolescent motherhood was 18.6%. Poverty and household crowding were associated with greater chances of AM (*p* values of 0.038 and <  0.001, respectively), as well as not being in a stable relationship (OR 2.26 (95%CI: 1.67, 3.07), *p* <  0.001). AM showed a greater chance of not using community health services (*p* <  0.001), had fewer prenatal consultations (β − 0.432 (95%CI: − 0.75, − 0.10)) and started prenatal care at a later date (β 0.38 (95%CI: 0.21, 0.55), *p* <  0.001)). AM are also less likely to be tested for HIV and more likely to have urinary tract infections.

**Conclusions:**

Interventions aimed at socially-vulnerable adolescents are suggested. However, if pregnant, adolescents should receive proactive and differentiated prenatal care.

## Introduction

Adolescent motherhood (AM) remains a matter of public health concern, especially in low-income and developing countries, where approximately 95% of this type of birth occurs [1]. It is associated with an increased incidence of maternal and particularly fetal complications, in addition to aggravating socioeconomic problems frequently observed in this age group [2].

Several factors are associated with AM, and among them lower access to public health services, greater social vulnerability, with lower income and level of schooling, according to a study published in 2018 by the Pan American Health Organization (PAHO), in partnership with the United Nations Children’s Fund (UNICEF) [3, 4]. Considering its impacts, AM was associated with an increased incidence of anemia, pre-eclampsia, urinary tract infection, low maternal weight gain, early weaning and low prenatal coverage, according to the literature [5]. Moreover, there are several negative socioeconomic effects for these adolescents, such as abandonment of educational activities, postponement of professional training and financial instability [6].

In Brazil, the AM rate was higher in the past, with a decrease occurring since the year 2000, but data from the Ministry of Health show that the number of children born to adolescent mothers in Brazil is one of the highest, when compared to Latin American and Caribbean countries, with 68.4 live births for every 1000 adolescents and young women (6.8%). In 2015, 18% of live births included children born to mothers aged up to 19 years, 0.9% of mothers aged 10 to 14 years and 17.3% of mothers aged 15 to 19 years [7].

Although several studies have been carried out on the events associated with AM, population evidence with representative samples, especially in low-to-middle-income countries (LMIC), is limited. The present study is a cross-sectional study of women aged 10 to 49 years living in the state of Ceará, Brazil. We assessed the prevalence of AM, as well as its association with Socioeconomic Factors and Obstetric Outcomes.

## Methods

### Study design and population

We analyzed data from the Maternal-Child Health Research in Ceará (PESMIC - *Pesquisa de Saúde Materno Infantil no Ceará)*, and full details on the methods can be found elsewhere [8]. The PESMIC is a population-based, cross-sectional study on maternal and child health of women aged 10 to 49 years, living in the state of Ceará, in northeastern Brazil. Ceará is one of the poorest states in Brazil, with a population of 9 million inhabitants living in a semiarid climate. Fortaleza (2.3 million inhabitants) is the capital city and urban commercial center of Ceará. The study area also included rural areas of Ceará, where subsistence farming is the predominant form of land use.

The PESMIC surveys used the cluster sampling method, based on the Brazilian Institute of Geography and Statistics (IBGE, *Instituto Brasileiro de Geografia e Estatística*) census tracts and stratification between the state capital Fortaleza, and the rural areas. The study was carried out from August to November 2017 and consisted of 160 randomly selected census tracts, which included a total of 3200 households. All women aged 10 to 49 years living in the households were included. Only women who reported at least one previous pregnancy were considered for the study analyses. Census tracts were constructed based on the division of each municipality into geographic areas of variable extensions, with a stable population of 300 families. To ensure the study population was representative, cities, census tracts and households were randomly selected. Once a census tract was defined and its corresponding map obtained, the location of the 20-house cluster to be investigated was determined. The cluster starting point (the first home to be visited) was randomly selected utilizing ArcGIS® software, GIS Inc. Households were visited consecutively in a counterclockwise fashion. Shops and abandoned buildings were excluded and substituted, and when the family was absent, up to three return visits were carried out in an attempt to obtain data.

### Measures

Information was obtained by trained interviewers about all female individuals in each household through reports and by checking the government’s pregnancy health booklet.

The definition of adolescence used in the study was the one used by the WHO [9, 10], and women who reported that their pregnancy occurred when they were up to 19 years of age were considered cases of adolescent motherhood.

In Brazil, all pregnant women receive the pregnancy health booklet, which contains health information about antenatal care and delivery recorded by the health professionals. These data were used to evaluate the woman’s prenatal received during pregnancy and the obstetric outcomes.

In addition to the assistance and obstetric variables, sociodemographic characteristics were also collected through self-report by the participants. Problems during breastfeeding (any) were self-reported by mothers following a direct interviewer question. Economic classification was assessed using the Brazil criterion, which classifies income into five strata, from A to E in decreasing order of income, a proxy scoring system for the consumption capacity of a household located in Brazilian metropolitan regions using the methodology of the statistical regression of the current family income logarithm, declared as a function of the number of home comfort items owned by the family and the level of schooling of the head of the household [11]. We also assessed food insecurity, using the Escala Brasileira de Insegurança Alimentar (EBIA), a validated tool with 15 items for assessing food insecurity [8].

### Statistical analysis

Descriptive statistics and AM prevalence rates adjusted for clustering by design are disclosed. Sample-adjusted logistic models with the calculation of unadjusted and adjusted odds ratios were used to determine the association between socioeconomic factors and AM, as well as the association between AM and obstetric outcomes, because in the first, AM would be the dependent variable of such factors, and in the second, AM would be the explanatory variable of such outcomes. Adjusted models that included selected covariates are shown, and a causal approach to multivariate analyses was used. Theoretical models were built, which considered sociocultural factors and poverty as the main determinants of AM, whereas others depict AM as a determinant of health care and obstetric outcomes. All study data were double-entered twice using EpiInfo 2000 and tested for concordance and the SPSS software, version 23 (SPSS Statistics for Windows, Version 23.0. IBM Inc.) was used in all analyses.

### Ethics

Written informed consent was obtained from participating women. Written consent for children was also given by mothers, and informed consent for adolescent minors was obtained from their parents or legal guardians. The PESMICs survey was approved by the Research Ethics Committee in Brazil (Research Ethics Committee of Unichrsitus University Center), under the number 73516417.4.0000.5049.

## Results

In total, 2.340 women were included in the analyzed sample, and the prevalence of adolescent motherhood (AM) was 18.6% (95% CI 17.1 to 20.2). The mean age at pregnancy of the 436 women who experienced adolescent motherhood was 16.8 ± 1.6 years and, on average, the pregnancy occurred 2.1 years before the interview. On average, the beginning of sexual activity for the women who experienced AM was 14.6 ± 1.7 years. Almost all of them were literate, most of them attended elementary school (53%) and reported a common-law marriage or formal marriage (58.9); 11% of them reported not following a religion. Almost 80% worked in household activities only, and only 5% reported smoking. The mean income reported was USD 304.04 ± 267.07, and 60% participated in or was registered to participate in the Brazilian income transfer program (*Bolsa Família*). Almost 90% of them belonged to social class E, the lowest, and more than 50% had food insecurity. (Table 1).

**Table 1:** Sociodemographic profile of women who experienced adolescent motherhood

	x̅ ± SD
Age (years)	19 ± 2.2
Age at last pregnancy (years)	16.8 ± 1.6
Time since last pregnancy	2.1 ± 1.6
Age at the first sexual intercourse (years)	14.6 ± 1.7
Age at the first pregnancy (years)	16.4 ± 1.8
Age at the birth of the first child (years)	17.1 ± 1.8
Number of people living in the household	4 ± 1.5
Family income (USD)	304.04 ± 267.07
	**n (%)**
Ethnicity	
White	55 (12.6)
Brown	349 (80.4)
Black	30 (6.9)
Level of schooling	
Elementary School	233 (53.6)
High School	129 (29.7)
College/University	72 (16.5)
Marital Status	
Single	166 (38)
Married	48 (11)
Common-law marriage	209 (47.9)
Separated	11 (2.5)
Widowed	2 (0.4)
Religion	
Catholic	262 (60.3)
Protestant/Evangelical	120 (27.6)
Spiritist	3 (0.6)
Other	1 (0.2)
None	48 (11)
Working status	
Yes, only at home	340 (78.5)
Yes, out of home	40 (9.2)
Yes, at home, with delivery	28 (6.4)
Does not work	25 (5.7)
Has private health insurance	
Yes, paid by the company	22 (5)
Yes, paid by the family	18 (4.1)
Does not have	395 (90.8)
Economic classification	
E	372 (87.1)
D, C, B and A	55 (12.9)

More than 95% of the interviews showed that the pregnant adolescents received essential prenatal care, such as iron and folic acid supplements, and were submitted to blood, urine, VDRL and HIV tests. However, 42.5% reported they were not aware of the family health program. On average, they started prenatal care at 2.6 ± 1.4 months of gestation and had a total of 7.9 ± 2.8 medical consultations. More than 10% reported having a miscarriage; 51% of AM deliveries were vaginal and 29.6% of the pregnant adolescents’ neonates were not breastfed shortly after birth. The AM showed a prevalence of several adverse postpartum conditions > 10%, including headache (26.8%), mastitis (29.8%), puerperal infection (18.9%) and breastfeeding problems (12.1%). (Table 2).

**Table 2:** Obstetric care profile of women who experienced adolescent motherhood

	n (%)
*Knows the PCS*?	
Yes, has been visited	144 (33.1)
Yes, CHS in the area but has not been visited	58 (13.3)
Yes, has heard about it	48 (11)
Does not know anything about it	185 (42.5)
*Miscarriage*	
Yes, in the last 12 months	21 (4.8)
Yes, more than 12 months ago	28 (6.4)
No	384 (88.6)
*Weight recorded in the prenatal booklet*	
Yes, in all the consultations	232 (61.8)
Yes, in some consultations	25 (6.6)
No	118 (31.4)
*Tests performed during prenatal care at the last pregnancy*	
Blood tests	390 (98.4)
Urinalysis	388 (97.9)
VDRL	379 (96.9)
HIV	379 (95.7)
Ultrasonography	390 (98.7)
*Type of delivery*	
Vaginal	200 (51)
Forceps	4 (1)
Caesarean section (for the 1st time)	166 (42.3)
Caesarean section (had already had a previous one)	22 (5.6)
*Problems after the delivery*	
Headache	106 (26.8)
Mastitis	118 (29.8)
Fever	75 (18.9)
Malodorous discharge	19 (4.8)
Fistula	20 (5)
Urinary infection	36 (9.1)
Arterial hypertension	25 (6.3)
Seizure	2 (0.5)
Bleeding	38 (9.6)
Breastfeeding problems	48 (12.1)
	**x̅ ± SD**
Number of prenatal consultations at the last pregnancy	7.9 ± 2.8
Month when prenatal care was started	2.6 ± 1.4

*PCS* Primary care services.

In the multivariate models of AM determining factors, it was observed that the younger the age of sexarche, the greater the chances of AM (OR 0.9 (95% CI: 0.83, 0.98), *p* value = 0.018). AM was also more often associated with not having a stable partner (OR 2.26 (95% CI: 1.67, 3.07), *p* <  0.001) and not following a religion (*p* = 0.011). Income and household crowding were also associated with AM, and households with higher income (*p* = 0.038) and less crowding (*p* <  0.001) showed lower chances of adolescent motherhood (Table 3).

**Table 3:** Socioeconomic factors associated with adolescent pregnancy

	Adolescent mothers					
	Yes	No				
	x̅ ± SD	x̅ ± SD	p_**a**_	OR (95%CI)_**a**_	p_**b**_	OR (95%CI)_**b**_
Age at the first sexual intercourse (years)	15 ± 1.7	17 ± 3.4	< 0.001*	–	0.018*	0.9 (0.83–0.98)
Age at the first pregnancy (years)	16 ± 1.8	22 ± 5.2	< 0.001*	–	< 0.001*	0.65 (0.6–0.69)
Age at the birth of the first child (years)	17 ± 1.8	22 ± 5.3	< 0.001*	–	c	
How many people live in the household?	4 ± 1.5	4 ± 1.3	< 0.001*	–	< 0.001*	0.77 (0.68–0.87)
Family income (Reais)	962 ± 845.2	1134 ± 1047.3	0.001*		0.038*	1 (1–1)
	**n (%)**	**n (%)**	**p**_**a**_	**OR (95%CI)**_**a**_	**p**_**b**_	**OR (95%CI)**_**b**_
*Ethnicity*						
White	55 (12,6)	359 (18,9)	0,005^*^	0,75 (0,48-1,18)	0,521	0,96 (0,49-1,86)
Brown	349 (80.4)	1385 (73.2)		1.24 (0.83–1.85)		1.17 (0.63–2.19)
Black	30 (6.9)	148 (7.8)		Ref.		
*Can read and write*?						
Yes	432 (99)	1831 (96.4)	0.014^*^	2.59 (0.85–7.91)	c	
No	1 (0.2)	34 (1.7)		0.32 (0.03–3.28)		
Can only sign their name	3 (0.6)	33 (1.7)		Ref.		
*Marital status*						
No partner	179 (41)	449 (23.6)	< 0.001*	2.24 (1.8–2.8)	< 0.001*	2.26 (1.67–3.07)
With partner	257 (58.9)	1450 (76.3)		Ref.		
*Religion*						
Yes	386 (88.9)	1798 (94.6)	< 0.001*	0.45 (0.32–0.64)	0.011*	0.48 (0.28–0.84)
None	48 (11)	102 (5.3)		Ref.		
*Region where you live*						
Capital/MRF	190 (43.6)	762 (40)	0.173	1.15 (0.92–1.44)	–	
Countryside	246 (56.4)	1142 (60)		Ref.		
*Has private health insurance*?						
Yes, paid by the company	22 (5)	129 (6.7)	0.001^*^	0.69 (0.43–1.09)	1	1.01 (0.43–2.33)
Yes, paid by the family	18 (4.1)	167 (8.7)		0.43 (0.27–0.7)		1 (0.55–1.8)
No	395 (90.8)	1602 (84.4)		Ref.		
*Food insecurity*						
No	168 (42.6)	726 (42.4)	0.954	1 (0.81–1.23)	–	–
Yes	226 (57.3)	983 (57.5)		Ref.		
*Social class*						
E	372 (87.1)	1407 (75.2)	< 0.001*	2.23 (1.66–2.99)	0.348	1.22 (0.8–1.86)
D, C, B and A	55 (12.8)	464 (24.7)		Ref.		

a – Unadjusted results. / b - Results adjusted for ethnicity, religion, marital status, social class, level of schooling, private health insurance, region where you live, number of people in the household, age at the first sexual intercourse, age at the first pregnancy, income and interviewer. / c - Not included in the final model due to multicollinearity.

*MRF* Metropolitan region of Fortaleza.

The multivariate models that assessed the impact of AM on obstetric outcomes showed that adolescent mothers had almost twice the chance of not being aware of the family health program (OR 1.8 (95% CI: 1.37, 2.36), *p* value < 0.001). It was also observed that adolescent mothers were more likely to have experienced a miscarriage in the last 12 months (OR 3.11 (95% CI: 1.69, 5.71), *p* < 0.001). Moreover, they had fewer prenatal consultations (β −0.432 (95% CI: −0.75, −0.10), *p*-value = 0.01), started prenatal care at a later date (β 0.38 (95% CI: 0.21–0.55), *p* < 0.001), and were less likely to be submitted to a C-section (OR 1.51 (95% CI: 1.2, 1.9), *p* < 0.001). Finally, pregnant adolescents are less likely to undergo an HIV screening test during pregnancy, and more likely to have puerperal infection (OR 1.36 (95% CI: 1.02–1.81), *p*-value = 0.034). (Table 4).

**Table 4:** Obstetric factors associated with pregnancy in adolescence

	Adolescent mothers					
	Yes	No				
	n (%)	n (%)	p_**a**_	OR (95%CI)_**a**_	p_**b**_	OR (95%CI)_**b**_
*Knows the PCS*?						
Yes, has been visited	144 (33.1)	750 (39.9)	< 0.001*	Ref.	< 0.001*	Ref.
Yes, PCS in the area but has not been visited	58 (13.3)	292 (15.5)		1.03 (0.74–1.43)		0.96 (0.68–1.35)
Yes, has heard about it	48 (11)	294 (15.6)		0.85 (0.59–1.2)		0.86 (0.59–1.23)
Does not know it	185 (42.5)	541 (28.8)		1.78 (1.36–2.33)		1.8 (1.37–2.36)
*Miscarriage*						
Yes, in the last 12 months	21 (4.8)	29 (1.5)	< 0.001*	3 (1.66–5.4)	< 0.001*	3.11 (1.69–5.71)
Yes, more than 12 months ago	28 (6.4)	272 (14.3)		0.42 (0.28–0.63)		0.43 (0.29–0.64)
No	384 (88.6)	1593 (84.1)		Ref.		Ref.
*Weight recorded in the prenatal booklet*						
Yes, in all consultations	232 (61.8)	1001 (64.4)	0.601	Ref.	–	–
Yes, in some consultations	25 (6.6)	89 (5.7)		1.21 (0.75–1.94)		
No	118 (31.4)	464 (29.8)		1.09 (0.85–1.4)		
*Tests performed in prenatal care during the last pregnancy*						
Blood tests						
Yes	390 (98.4)	1619 (99.1)	0.235^b^	0.56 (0.25–1.24)	–	–
No	6 (1.5)	14 (0.8)		Ref.		
Urinalysis						
Yes	388 (97.9)	1613 (98.6)	0.318	0.66 (0.31–1.37)	–	–
No	8 (2)	22 (1.3)		Ref.		
VDRL						
Yes	379 (96.9)	1602 (98.2)	0.085	0.55 (0.3–1)	–	–
No	12 (3)	28 (1.7)		Ref.		
HIV						
Yes	379 (95.7)	1604 (98.1)	0.004^*^	0.41 (0.24–0.72)	.004^*^	0,42 (0,23-0,76)
No	17 (4.2)	30 (1.8)		Ref.		Ref.
Ultrasonography						
Yes	390 (98.7)	1616 (98.9)	0.699^b^	0.82 (0.35–1.89)	–	–
No	5 (1.2)	17 (1)		Ref.		
*Type of delivery*						
Vaginal	200 (51)	635 (39.2)	< 0.001*	1.63 (1.29–2.04)	0.001	1,51 (1,2-1,9)
Forceps	4 (1)	10 (0.6)		2.07 (0.64–6.61)		2,3 (0,69-7,62)
Caesarean section	188 (47.9)	973 (60.1)		Ref.		Ref.
*Baby was breastfed within the 1st hour after birth?*						
Yes	278 (70.3)	1190 (73)	0.294	0.87 (0.68–1.12)	–	–
No	117 (29.6)	440 (26.9)		Ref.		
*Problems after delivery*						
Headache						
Yes	106 (26.8)	432 (26.4)	0.862	1.02 (0.78–1.33)	–	–
No	289 (73.1)	1204 (73.5)		Ref.		
Mastitis						
Yes	118 (29.8)	430 (26.2)	0.147	1.19 (0.93–1.52)	–	–
No	277 (70.1)	1207 (73.7)		Ref.		
Fever						
Yes	75 (18.9)	239 (14.5)	0.030^*^	1.37 (1.03–1.81)	0.034*	1.36 (1.02–1.81)
No	320 (81)	1399 (85.4)		Ref.		Ref.
Malodorous discharge						
Yes	19 (4.8)	62 (3.7)	0.345	1.28 (0.72–2.28)	–	–
No	375 (95.1)	1576 (96.2)		Ref.		
Fistula						
Yes	20 (5)	68 (4.1)	0.418	1.23 (0.72–2.1)	–	–
No	374 (94.9)	1570 (95.8)		Ref.		
Urinary infection						
Yes	36 (9.1)	112 (6.8)	0.115	1.37 (0.92–2.03)	–	–
No	358 (90.8)	1526 (93.1)		Ref.		
Arterial hypertension						
Yes	25 (6.3)	135 (8.2)	0.204	0.75 (0.49–1.13)	–	–
No	370 (93.6)	1502 (91.7)		Ref.		
Seizure						
Yes	2 (0.5)	20 (1.2)	.217^b^	0.41 (0.09–1.8)	–	–
No	393 (99.4)	1617 (98.7)		Ref.		
Breastfeeding problems						
Yes	48 (12.1)	148 (9)	0.061	1.38 (0.97–1.97)	–	–
No	347 (87.8)	1487 (90.9)		Ref.		
Bleeding						
Yes	38 (9.6)	118 (7.2)	0.102	1.37 (0.91–2.05)	–	–
No	356 (90.3)	1520 (92.7)		Ref.		
	**x̅ ± SD**	**x̅ ± SD**	**p**_**a**_	**OR (95%CI)**_**a**_	**p**_**b**_	**β**_**b**_
Number of prenatal consultations at the last pregnancy	8 ± 2.8	8 ± 2.9	0.002*	–	0.010	−0.432 (−0.75. -0.10)
Month when prenatal care was started	3 ± 1.4	2 ± 1.5	< 0.001*	–	< 0.001*	0.38 (0.21–0.55)

a - Unadjusted results. / b - Results adjusted by income.

*PCS* Primary care services.

## Discussion

This study found a prevalence of AM of 18.6% in the state of Ceará, Brazil. Moreover, it identified the following significant sociodemographic factors associated with AM: lower age at sexarche, higher level of schooling, not having a stable partner, not following a religion, lower family income, higher number of people living in the household. We also identified the obstetric factors for AM compared to adult pregnant women, such as not being aware of the Family Health Program, reporting a miscarriage in the last 12 months, fewer prenatal consultations, less HIV testing during pregnancy and occurrence of puerperal infection. Our study showed a higher prevalence of AM in comparison to the mean worldwide rate (11.6%) [12] and higher than that found in most countries in Latin America and the Caribbean (15%) [13], equal to the Brazilian prevalence in 2015 [7]. The prevalence found in the present study is similar to that of African countries (18.8%) [14]. In Latin America and the Caribbean, the pregnancy rate is the second highest in the world, second only to the one found in the Sub-Saharan Africa [13].

Regarding the socioeconomic factors, in our study, AM was more often associated with a higher degree of schooling, dissimilar from what is often observed in population-based studies, even in developed countries [15]. The literature also indicates that adolescents who did not abandon school are less likely to get pregnant again [16], indicating the need to invest in social support to prevent school abandonment by the adolescents after pregnancy. Regarding this aspect, the Pan-American Health Organization indicates that there is no homogeneity in the profile of pregnant adolescents between countries in Latin America and the Caribbean. However, some factors such as poverty, low level of schooling, social exclusion, unequal social norms and racism stand out [13]. Hence, the higher the HDI, the lower the rate of infants born to adolescent mothers [17]. This is in agreement with the present study, which showed that higher income and lower household crowding were protective factors against AM.

The predominance of vaginal delivery among the adolescents is in agreement with most studies in the literature [18–20]. In the present study, the pregnant adolescents were 1.5 times more likely to undergo vaginal delivery when compared to the adult pregnant women. It should be noted that the choice of delivery method depends on a variety of factors, such as the woman’s wishes and the obstetrician’s policy [18]. In Brazil, there has been a progressive incentive to increase the number of vaginal births since 2011, with the advent of the *Projeto Rede Cegonha (*The Stork Network Project), increased in 2014 with *Projeto do Parto Adequado* (Adequate Childbirth Project), aiming to prevent unnecessary C-section births [21, 22].

Although most women did not have private health insurance, which means they were treated by the Brazilian Public Health System, SUS (*Sistema Único de Saúde*), 42.5% of the adolescents reported not being aware of the Family Health Program, and the adolescents showed a two-fold chance of not having received that information. Although the relevance of the Family Health Strategy actions aimed at adolescents is important to decrease their vulnerabilities through comprehensive care, the low level of awareness found in this age group and little offer of actions aimed at this population leaves something to be desired in primary health care. This may reflect the lack of knowledge about the family health strategy indicated in our study by the group of pregnant adolescents. At the global level, due to population heterogeneity and the assessed objectives, the information does not allow us to provide exact numbers regarding the percentage of use of health services by adolescents, but some studies mention that improving the reasons that discourage adolescents from seeking health care at the basic health unit will lead to improvements in care and, consequently, better outcomes [23, 24]. A study carried out in Canada identified that pregnant adolescents attend prenatal consultations more often than the overall average among adult pregnant women. In that country, the PA rate is 2.9%, according to the MES (Maternity Experience Survey) study and pregnant adolescents attend 2.5 times more prenatal consultations than non-adolescent pregnant women [25].

In our study, although the adolescents started the prenatal care at a later date than the adult pregnant women, the first consultation took place in the first trimester, as recommended by the national guidelines. Over the years, the family structure has undergone reformulations, changing the rigid format of little dialogue between parents and teenagers, and focusing on the father as an agent of repression. However, difficulties remain regarding conversations about sexuality. The association of the adolescents’ greater sexual freedom with a still restricted dialogue favors the fact that, when facing a first pregnancy at an early stage of their reproductive age, the adolescent is afraid of retaliation and hides the diagnosis. At this time, the family health strategy should be a major factor for adolescent care [26].

Regarding this finding, the literature shows that adolescents can hide their pregnancy and thus start prenatal care at a later date, alerting to the need to investigate vague and non-specific pregnancy-related complaints in this group [16]. It is worth mentioning that the late start of prenatal care may lead to a lower number of consultations, which may justify the fact that pregnant adolescents in this study had fewer consultations when compared to the adult women.

This study showed a higher chance of miscarriage in the previous 12 months among pregnant adolescents, a result similar to another study that found a higher risk of miscarriage and stillbirth in this population, when compared to adult pregnant women [27]. One explanation for this result would be the possibility that pregnant women in a situation of social vulnerability, which is a characteristic of our sample, will seek the resolution of pregnancy through unsafe abortion. A systematic review published in 2020 on unsafe abortions in Brazil concludes that women who are more vulnerable socially and living in less developed regions more often resort to this type of abortion [28].

The only significant postpartum obstetric outcome was the higher occurrence of puerperal infection in adolescents, based on the report of fever in the postpartum period. According to the Pan-American Health Organization [29], pregnant adolescents are at increased risk for systemic infections and endometritis. This outcome is seldom reported in the literature, with greater emphasis on other adverse results, such as anemia, pre-eclampsia, urinary tract infection, and gestational diabetes [16, 18, 27, 30]^.^

### Limitations

One aspect that limited the discussion of this study results was the fact that the studies available in the literature are heterogeneous, both regarding the classification of adolescence and the age range of the analyzed sample, hindering a homogeneous comparison. Another issue that deserves to be highlighted is the diversity of cultural contexts regarding adolescent pregnancy, which involves the conscious or unconscious decision of the pregnant adolescent to have children. The cross-sectional design is also important, as it prevents making causal inferences about the investigated factors. Finally, the retrospective nature and the theoretical foundation based only on the variables evaluated in PESMIC, which did not allow, for instance, analyzing situations such as repeated adolescent pregnancy, especially within 2 years after the first child.

## Conclusions

Adolescents need to be educated about contraception and safe sex, aiming to reduce pregnancy at this stage of life, as well as sexually transmitted infections. However, if pregnant, adolescents should receive specialized prenatal care and assistance focused on the reduction of risk of complications and to better prepare this binomial and the existence of the new family. The need to reinforce intersectoral actions, such as the Health at School Program (*Programa Saúde na Escola*), is highlighted, aiming to increase the level of knowledge about the assistance offered in primary health care. Moreover, it is necessary to guide pregnant women during prenatal care about the actions developed at this level of health care, aiming to encourage comprehensive assistance. The need for protection against repeated pregnancies also deserves attention, as the literature indicates a direct association with both maternal and neonatal complications, such as hemorrhagic syndrome and preterm delivery [31]. It is worth mentioning the importance of age when becoming pregnant, and the fact that a first pregnancy under the age of 15 years seems to entail a higher risk of complications. Other social determinants, such as the lack of a partner, the low level of schooling and the lack of plans for the future are factors that contribute to adverse pregnancy outcomes [1].

For pregnant adolescents, family support and individualized assistance during pregnancy also bring beneficial results for adequate childbirth and good child development. Considering all these facts, studies directly related to adolescents can be essential to establish the determinants of adverse outcomes of adolescent motherhood.

## Data Availability

The datasets used and/or analyzed during the current study are available from the corresponding author on reasonable request.

## Abbreviations

PESMIC: Pesquisa de saúde materno infantil do Ceará
IBGE: Brazilian institute of geography and statistics
USDA: United States department of agriculture
ANC: Antenatal care
LMIC: Low- and middle-income countries
SES: Socioeconomic status
SDG: Sustainable development goals
